# Olaparib Is a Mitochondrial Complex I Inhibitor That Kills Temozolomide-Resistant Human Glioblastoma Cells

**DOI:** 10.3390/ijms222111938

**Published:** 2021-11-03

**Authors:** Luca X. Zampieri, Martina Sboarina, Andrea Cacace, Debora Grasso, Léopold Thabault, Loïc Hamelin, Thibaut Vazeille, Elodie Dumon, Rodrigue Rossignol, Raphaël Frédérick, Etienne Sonveaux, Florence Lefranc, Pierre Sonveaux

**Affiliations:** 1 Pole of Pharmacology and Therapeutics, Institut de Recherche Expérimentale et Clinique (IREC), Université Catholique de Louvain (UCLouvain), 1200 Brussels, Belgium; luca.zampieri@uclouvain.be (L.X.Z.); marty.sboa@gmail.com (M.S.); andre.cacace@libero.it (A.C.); debora89grasso@gmail.com (D.G.); leopold.thabault@uclouvain.be (L.T.); loic.hamelin@uclouvain.be (L.H.); thibaut.vazeille@uclouvain.be (T.V.); 2 Louvain Drug Research Institute (LDRI), UCLouvain, 1200 Brussels, Belgium; raphael.frederick@uclouvain.be (R.F.); esonveaux@hotmail.com (E.S.); 3 INSERM U1211, Laboratory of Rare Diseases, Metabolism and Genetics (MRGM), Ecole des Sages Femmes, Bordeaux University, 33076 Bordeaux, France; elodie.dumon@chu-bordeaux.fr (E.D.); rodrigue.rossignol@u-bordeaux.fr (R.R.); 4 Service de Neurochirurgie, Hôpital Erasme, Université Libre de Bruxelles (ULB), 1070 Brussels, Belgium; florence.lefranc@erasme.ulb.ac.be

**Keywords:** glioblastoma, chemoresistance, temozolomide (TMZ), cancer metabolism, mitochondria, PARP inhibitors, metformin

## Abstract

Glioblastoma represents the highest grade of brain tumors. Despite maximal resection surgery associated with radiotherapy and concomitant followed by adjuvant chemotherapy with temozolomide (TMZ), patients have a very poor prognosis due to the rapid recurrence and the acquisition of resistance to TMZ. Here, initially considering that TMZ is a prodrug whose activation is pH-dependent, we explored the contribution of glioblastoma cell metabolism to TMZ resistance. Using isogenic TMZ-sensitive and TMZ-resistant human glioblastoma cells, we report that the expression of O6-methylguanine DNA methyltransferase (MGMT), which is known to repair TMZ-induced DNA methylation, does not primarily account for TMZ resistance. Rather, fitter mitochondria in TMZ-resistant glioblastoma cells are a direct cause of chemoresistance that can be targeted by inhibiting oxidative phosphorylation and/or autophagy/mitophagy. Unexpectedly, we found that PARP inhibitor olaparib, but not talazoparib, is also a mitochondrial Complex I inhibitor. Hence, we propose that the anticancer activities of olaparib in glioblastoma and other cancer types combine DNA repair inhibition and impairment of cancer cell respiration.

## 1. Introduction

Glioblastomas (GBMs) account for ~50% of all brain tumors in humans [1]. They represent the ultimate grade of brain cancer and are associated with a very poor prognosis, with a mean survival time of only a few months despite surgery, radiotherapy, and chemotherapy [2,3]. The current standardized clinical protocol includes maximal surgical resection followed by radiotherapy and concomitant followed by adjuvant temozolomide (TMZ) chemotherapy [4]. This protocol has enabled the five-year survival of GBM patients to increase from ~2% to ~10% [2]. However, GBMs continue to have a poor prognosis, which is mainly due to three biological characteristics: they diffusely invade distant brain tissue through multiple single migrating cells [5], which limits the benefit of surgical resection; they can acquire resistance to TMZ; and they most often recur [6].

TMZ is an orally available alkylating agent that was initially developed for brain cancer therapy based on its ability to cross the blood-brain barrier (BBB) [7]. It is a prodrug that requires a two-step conversion: it is first hydrolyzed to form intermediate compound monomethyl-triazeno-imidazole-carboxamide (MTIC), which then breaks down to form the reactive methyldiazonium ion [8]. This conversion is pH-dependent and does not require enzymatic activation: TMZ is stable/inactive at acidic pH but decomposes to MTIC at pH > 7; and MTIC is stable/inactive at alkaline pH, but fragments to produce a methyldiazonium ion at pH < 7 [9,10] (Figure S1a). When produced intracellularly, methyldiazonium methylates DNA at O6-guanine residues (O6-MeG) in guanine-rich regions, as well as N7-guanine (N7-MeG) and N3-adenine (N3-MeA) [10,11]. O6-methylguanine methylation is the primary cause of TMZ-induced cytotoxicity.

Resistance to TMZ can occur when the gene promoter of O6-methylguanine DNA methyltransferase (MGMT) is demethylated, thus inducing the expression of the MGMT enzyme that specifically demethylates O6-MeG in DNA [12]. A positive correlation between MGMT promoter methylation and improved survival was first demonstrated in uncontrolled studies of GBM patients who were treated with nitrosoureas [13] or with TMZ [14]. In a randomized study, Hegi et al. [12] further evidenced that patients whose tumors were methylated at the MGMT promoter had a higher survival benefit from combined radiotherapy and TMZ chemotherapy compared to patients with an unmethylated MGMT promoter. Consequently, MGMT promoter methylation was proposed as a prognostic biomarker of the response of GBMs to TMZ [15,16], and quality assured MGMT testing was implemented as a molecular diagnostic method in the 2016 World Health Organization (WHO) classification of brain tumors [17]. However, a Phase II clinical trial showed no clinical benefit of combining TMZ with the MGMT inhibitor O6-benzylguanine (O6BG) in recurrent TMZ-resistant GBM [18], suggesting that additional mechanisms may account for GBM resistance to TMZ chemotherapy.

Similar to other tumor types, GBMs are highly heterogeneous in their cellular composition and metabolic activities, and these heterogeneities vary over the course of the disease and its treatment [19,20]. We have previously shown that long-term treatment of two human GBM cell lines of astrocytic origin, U373 and T98G, with TMZ induced the expression of the glucose transporter GLUT3 [21], thus suggesting that acquired resistance to TMZ could be associated with a switch to a more glycolytic metabolism. Because TMZ, as a prodrug, is activated in a pH-dependent manner [9,10] and a glycolytic metabolism acidifies extracellular pH (pHe) with lactic acid, this in vitro study aimed to test whether metabolic reprogramming could participate in GBM resistance to TMZ. It further aimed to test whether targeting selected metabolic changes could have a therapeutic value against TMZ-resistant human GBM cells.

## 2. Results

### 2.1. Selection of Temozolomide-Resistant Human Glioblastoma Cells

To determine whether metabolic changes could account for acquired resistance to TMZ, we first aimed to generate isogenic models of TMZ-sensitive and TMZ-resistant human glioblastoma cells. U373 MG and T98G cells were treated for cycles of 72 h with increasing doses of TMZ (from 0.1 µM to 150 µM), with successive increments of 1.5-fold allowing recovery between doses [21], yielding U373-R and T98G-R cells, respectively. Filiation was authenticated by short tandem repeat (STR) profiling (Table S1). U373-R and T98G-R were 97.96% and 98.04% coincident with parental U373 and T98G cell lines, respectively.

**Figure 1:**
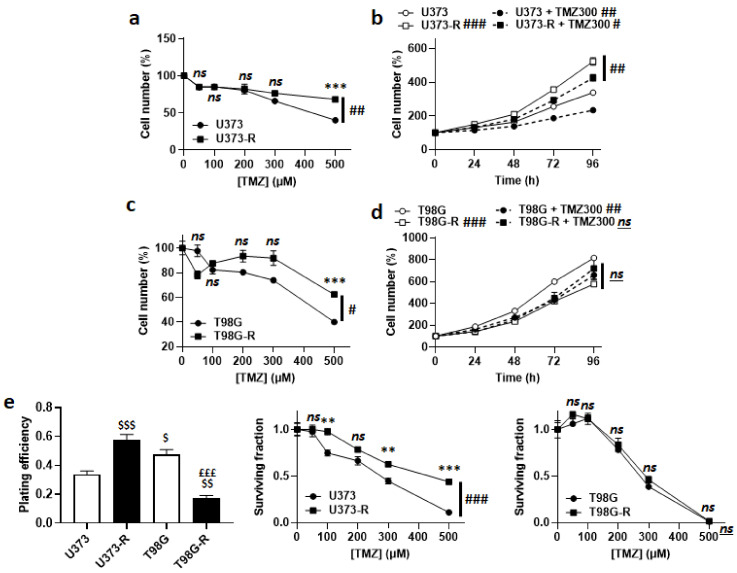
Validation of U373/U373-R as an isogenic model of TMZ-sensitive/resistant human glioblastoma cells. (**a**) Number of U373 and U373-R cells 72 h after treatment with the indicated concentrations of TMZ. For each curve, data are normalized to the number of the corresponding vehicle-treated cells (*n* = 4). (**b**) Number of U373 and U373-R glioblastoma cells that were treated ± 300 µM temozolomide (TMZ300) determined over time. For each curve, data are normalized to the number of corresponding cells at time 0 (*n* = 4). (**c**) As in (**a**), but for T98G and T98G-R glioblastoma cells (*n* = 4). (**d**) As in (**b**), but for T98G and T98G-R cells (*n* = 4). (**e**) Clonogenic assays of U373, U373-R, T98G, and T98G-R cells that were pretreated for 72 h with increasing concentrations of TMZ. Displayed are the plating efficiency of vehicle-treated cells (left; *n* = 6); the surviving fraction comparing U373 with U373-R cells (middle; *n* = 6); and the surviving fraction comparing T98G with T98G-R cells (right; *n* = 6), where the data are normalized to the number of corresponding vehicle-treated cells. All data are shown as means ± SEM. ** *p* < 0.01, *** *p* < 0.005, ns: *p* > 0.05 versus the corresponding wild-type cells; ^#^ *p* < 0.05, ^##^ *p* < 0.01, ^###^ *p* < 0.005, ns: *p* > 0.05 for whole curve comparison; ^$^ *p* < 0.05, ^$$^ *p* < 0.01, ^$$$^ *p* < 0.005 compared to the vehicle-treated U373 cells; ^£££^ *p* < 0.005 compared to the vehicle-treated T98G cells; by two-way ANOVA with Sidak’s post hoc test (**a**–**d**,**e** middle, **e** right) or one-way ANOVA with Tukey’s post hoc test (**e** left).

Acquired TMZ resistance was first verified by treating the cells for 72 h with doses of TMZ from 0 to 500 µM. Direct cell counting revealed that U373-R were more resistant to TMZ than U373 cells (Figure 1a), despite a faster proliferation rate both in the absence and in the presence of 300 µM TMZ (Figure 1b). Resistance to TMZ was not complete, as U373-R cell replication was still significantly slowed-down by TMZ. Similarly, compared to T98G, T98G-R cells were significantly preserved when they were exposed to increasing doses of TMZ (Figure 1c). T98G-R had a slower proliferation rate than T98G cells, which was unaffected by 300 µM TMZ (Figure 1d).

In clonogenic assays, U373-R had a better plating efficiency (PE) than U373 cells, with confirmed clonogenic resistance to increasing doses of TMZ (Figure 1e and Table S2). T98G-R had a worse PE than T98G cells, with no difference in clonogenic resistance (Figure 1e and Table S2). These data validated the U373/U373-R model, but not the T98G/T98G-R model. To maintain their resistant phenotype, U373-R cells were weekly exposed to 150 µM TMZ for 72 h.

### 2.2. High MGMT Expression Does Not Account for the Acquired Resistance of Human U373-R Glioblastoma Cells to TMZ

**Figure 2:**
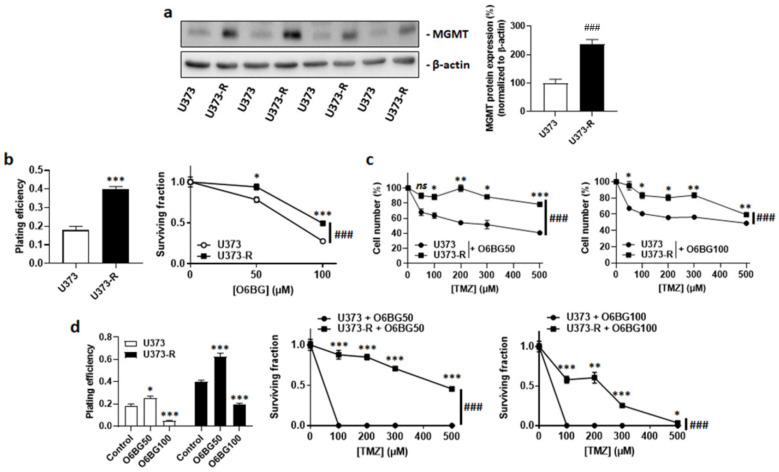
High MGMT protein expression does not account for TMZ resistance in the U373/U373-R isogenic human glioblastoma model. (**a**) O6-methylguanine DNA methyltransferase (MGMT) and β-actin (loading control) protein expression as determined by Western blotting in U373 and U373-R glioblastoma cells in the absence of TMZ, with pictures on the left and quantification on the right (*n* = 4). (**b**) Clonogenic assays of the cells that were pretreated for 72 h with increasing concentrations of MGMT inhibitor O6-benzylguanine (O6BG). Displayed are the plating efficiency of the vehicle-treated cells at 72 h (left; *n* = 6); and the surviving fraction (right; *n* = 6–12), where the data are normalized to the number of corresponding vehicle-treated cells. (**c**) Number of cells 72 h after treatment with the indicated concentrations of TMZ in combination with 50 µM O6BG (O6BG50; left; *n* = 4) or 100 µM O6BG (O6BG100; right; *n* = 4). (**d**) Clonogenic assays of the cells that were pretreated for 72 h with increasing concentrations of TMZ and O6BG. Displayed are the plating efficiency of the cells that were treated ± O6BG (left; *n* = 6); and the surviving fractions of the cells that received O6BG50 (middle; *n* = 6) or O6BG100 (right; *n* = 5–6), where the data are normalized to the number of corresponding vehicle-treated cells. All data are shown as means ± SEM. * *p* < 0.05, ** *p* < 0.01, *** *p* < 0.005, ns: *p* > 0.05 versus the corresponding control; ^###^ *p* < 0.005 for whole curve comparison; by Student’s *t*-test (**a**,**b** left), two-way ANOVA with Sidak’s post hoc test (**b** right, **c**,**d** middle, **d** right), or one-way ANOVA with Dunnett’s post hoc test (**d** left).

MGMT dealkylates DNA, which can confer resistance to TMZ [12,22]. Isogenic cell comparison revealed that, even in the absence of TMZ, U373-R had increased MGMT protein expression compared to U373 cells (Figure 2a). The MGMT inhibitor O6BG used at clinically relevant concentrations of 50 µM or 100 µM [23] reduced the clonogenic survival of both U373 and U373-R cells (Figure 2b). However, it did not selectively restore the sensitivity of U373-R cells to TMZ, which was evidenced by direct cell counting (Figure 2c) and clonogenic survival assays (Figure 2d and Table S2). While 100 µM O6BG reduced the PE of both U373 and U373-R, U373 cells with a low MGMT expression responded better to O6BG + TMZ than to TMZ alone (compare Figure 1e and Figure 2d, and see Table S2).

### 2.3. Extracellular Acidification Inactivates TMZ, but This Metabolic Strategy Is Not Used by Human U373-R Glioblastoma Cells to Resist to TMZ

**Figure 3:**
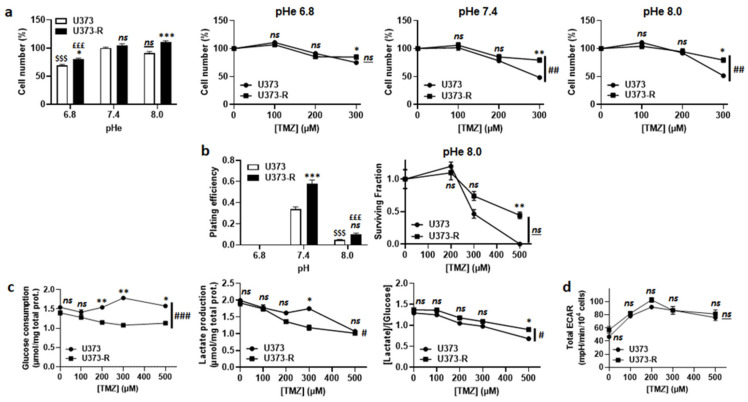
Extracellular pH alkalization increases U373-R sensitivity to TMZ, but resistant cells do not acidify the extracellular milieu. (**a**) Number of U373 and U373-R glioblastoma cells after 72 h of culture at different extracellular pH (pHe) ± TMZ. Shown are vehicle-treated cells at different pHe (far left); and the cells at pHe 6.8 (middle left), pHe 7.4 (middle right), or pHe 8.0 (far right) that were treated with increasing concentrations of TMZ (*n* = 4 all). (**b**) Clonogenic assays of cells after 72 h of culture at different pHe ± TMZ. Displayed are the plating efficiency of untreated cells (left; *n* = 5–6); and the surviving fraction of cells at pHe 8.0 (right; *n* = 5–6), where the data are normalized to the number of the corresponding vehicle-treated cells. (**c**) Glucose consumption (left) and lactate release (right) that were measured using an ISCUSflex CMA600 enzymatic analyzer after 72 h of cell culture with increasing concentrations of TMZ. The right graph represents the glycolytic efficiency ([lactate]/[glucose]). All data were normalized to total protein contents (*n* = 3 all). (**d**) The cells were pretreated for 72 h with increasing concentrations of TMZ, after which their glycolytic extracellular acidification rates (ECARs) were measured on a Seahorse XF96 bioenergetic analyzer (*n* = 17–24). All data are shown as means ± SEM. * *p* < 0.05, ** *p* < 0.01, *** *p* < 0.005, ns: *p* > 0.05 versus corresponding U373 cells; ^#^ *p* < 0.05, ^###^ *p* < 0.01, ^###^ *p* < 0.005, ns: *p* > 0.05 for whole curve comparison; ^$$$^ *p* < 0.005, ns: *p* > 0.05 versus U373 cells at pH 7.4; ^£££^ *p* < 0.005 versus U373-R cells at pH 7.4; by one-way ANOVA with Tukey’s post hoc test (**a** far left, **b** left) or two-way ANOVA with Sidak’s post hoc test (**a** middle left, **a** middle right, **a** far right, **b** right, **c**,**d**).

TMZ is a prodrug with pH-dependent activation [8,10] (Figure S1a). According to the theory, TMZ lost its anticancer activity following acidification of the extracellular pH (pHe) from 7.4 to 6.8 (Figure 3a). However, switching pHe from 7.4 to 8.0 did not sensitize U373-R cells to TMZ. In the absence of TMZ, clonogenic assays revealed a complete loss of clonogenicity of both of the cell variants at pHe 6.8 and a strong reduction at pHe 8.0 compared to pHe 7.4 (Figure 3b and Table S2). In the presence of TMZ at pHe 8.0, U373-R nevertheless kept a clonogenic advantage over U373 cells. These data showed that extracellular acidification inactivates TMZ but it is also detrimental to clonogenicity per se. Hence, even if U373-R were slightly more glycolytic than U373 cells (Figure 3c), measurements of the glycolytic extracellular acidification rates (ECARs) in unbuffered medium using the Seahorse technology revealed that U373-R cells did not actively modulate pHe to resist to TMZ (Figure 3d). Interestingly, however, treating U373 or U373-R cells with 100 µM O6BG induced a glycolytic switch in both variants (Figure S1b) that was associated to a dose-dependent increase in total ECAR with a net advantage for U373-R cells (Figure S1c). Inhibition of TMZ activation at low pHe could thus explain, at least in part, why U373-R cells are not sensitized to TMZ by O6BG.

### 2.4. Maintenance of Fitter Mitochondria with Enhanced Oxidative Activity Participates in the Resistance of Human U373-R Glioblastoma Cells to TMZ

**Figure 4:**
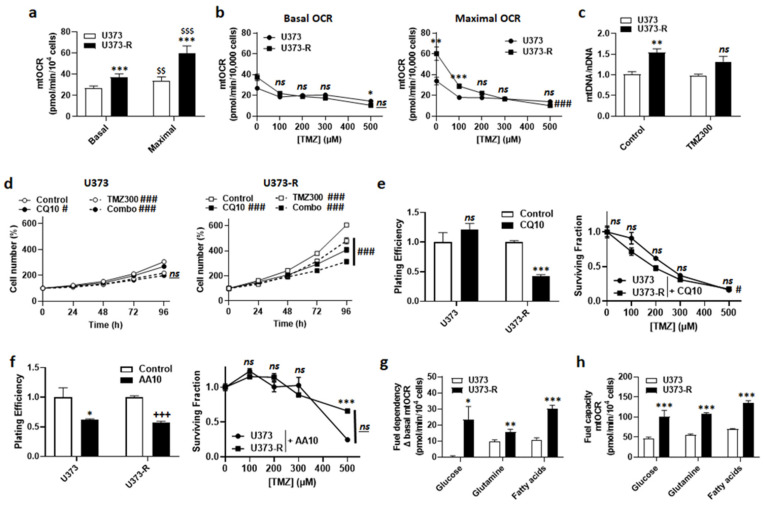
Fitter, more oxidative mitochondria participate in the resistance of U373-R cells to TMZ. (**a**) The basal and maximal mitochondrial oxygen consumption rates (mtOCRs) of U373 and U373-R glioblastoma cells were determined on a Seahorse XF96 bioenergetic analyzer (*n* = 6). (**b**) The cells were treated for 72 h with increasing doses of TMZ, after which basal mtOCRs (left) and maximal mtOCRs (right) were measured (*n* = 14–24). (**c**) The cells were treated for 72 h ± TMZ300, after which the mitochondrial DNA/nuclear DNA ratios (mtDNA/nDNA) were determined using RT-qPCR (*n* = 6). (**d**) Number of U373 (left; *n* = 12–16) and U373-R (right; *n* = 12–16) cells over time upon treatment ± 10 µM of autophagy inhibitor chloroquine (CQ10) ± TMZ300. (**e**) Clonogenic assays of U373 and U373-R cells that were pretreated for 72 h with increasing concentrations of TMZ ± 10 µM chloroquine (CQ10). Displayed are the plating efficiency of TMZ-untreated cells (left; *n* = 3–5); and the surviving fractions (right; *n* = 6), where the data are normalized to the number of corresponding TMZ-untreated cells. (**f**) As in (**e**) but using 10 µM of ETC Complex III inhibitor antimycin A (*n* = 3–6). (**g**) The glucose, glutamine, and fatty acid fuel dependency of U373 and U373-R cells that was determined on a Seahorse XF96 bioenergetic analyzer (*n* = 6–8). (**h**) The glucose, glutamine, and fatty acid fuel capacity of U373 and U373-R cells that was determined on a Seahorse XF96 bioenergetic analyzer (*n* = 4–8). All data are shown as means ± SEM. * *p* < 0.05, ** *p* < 0.01, *** *p* < 0.005, ns: *p* > 0.05 versus the corresponding U373 cells; ^#^ *p* < 0.05, ^###^ *p* < 0.005, ns: *p* > 0.05 for whole curve comparison; ^$$^ *p* < 0.01, ^$$$^ *p* < 0.005 versus U373 basal; ^+++^ *p* < 0.005 versus untreated U373-R; by one-way ANOVA with Tukey’s post hoc test (**a**), two-way ANOVA with Sidak’s post hoc test (**b**,**d**,**e** right, **f** right) or Student’s *t*-test (**c**,**e** left, **f** left, **g**,**h**).

In general, mitochondrial activities play a role in acquired chemo- and radio-resistance [24,25]. We therefore explored the potential contribution of oxidative phosphorylation (OXPHOS) to TMZ resistance in our model. In the absence of TMZ, U373-R cells had higher basal and maximal mtOCRs than U373 cells (Figure 4a). TMZ dose-dependently reduced mtOCRs in both variants (Figure 4b), indicating that it acts as a mitochondrial poison. Accordingly, even if U373-R initially maintained a higher maximal mtOCR up to 100 µM TMZ, mtOCR then decreased down to the level of U373 cells (Figure 4b). Without TMZ, the mitochondrial DNA/nuclear DNA ratio (mtDNA/nDNA) of U373-R was higher than that of U373 cells (Figure 4c). This difference was lost upon treatment with 300 µM TMZ.

An increased mitochondrial turnover rate (i.e., increased mitophagy to eliminate damaged mitochondria + increased mitochondrial biogenesis to repopulate the cells with intact mitochondria) has previously been suggested to participate in acquired cancer cell resistance to chemo- and radio-therapy [24,25,26]. This is a typical feature of cancer stem cells [27,28]. Inhibiting autophagy/mitophagy with 10 µM chloroquine (CQ), an agent that increases lysosomal pH, had no effect on U373 but reduced U373-R cell numbers either in the absence or in the presence of 300 µM TMZ (Figure 4d). Comparatively, 10 µM bafilomycin A1, a vacuolar ATPase (V-ATPase) inhibitor, sensitized both U373 and U373-R cells to 150 µM TMZ (Figure S2). CQ selectively reduced the PE and restored the clonogenic sensitivity of U373-R cells to TMZ (Figure 4e and Table S2).

U373-R cells with fitter mitochondria had a better mtOCR than U373 cells (Figure 4a). We therefore tested whether elevated OXPHOS directly participates in TMZ chemoresistance. Although inhibiting ETC Complex III with 10 µM antimycin A selectively reduced the PE of both U373 and U373-R cells, it actually increased the clonogenic survival of both variants to TMZ (Figure 4f and Table S2). Without TMZ, U373 cell respiration depended on glutamine and fatty acids, but not on glucose anaplerosis (Figure 4g). U373-R cells rather used the three fuels simultaneously, demonstrating increased anaplerotic plasticity. Interestingly, U373 and U373-R had the capacity to use either of the three oxidative fuels in case of inhibition of the two alternative anaplerotic pathways (Figure 4h), U373-R cells were always more efficient (Figure 4g–h), explaining their higher OXPHOS activity.

Together, these data indicated that acquired TMZ resistance in the U373/U373-R model is caused, at least in part, by the maintenance of fitter mitochondria that were characterized by enhanced anaplerotic versatility.

### 2.5. Olaparib Is an ETC Complex I Inhibitor That Sensitizes Human U373-R Glioblastoma Cells to TMZ

**Figure 5:**
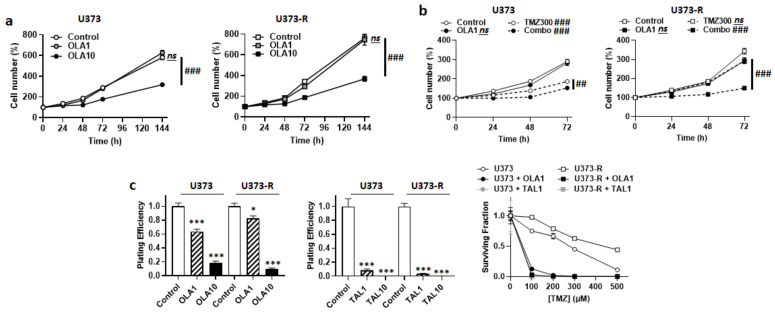
PARP inhibitors sensitize U373 and U373-R cells to TMZ. (**a**) Number of U373 (left; *n* = 4–8) and U373-R (right; *n* = 4–7) glioblastoma cells that were treated ± 1 µM of PARP inhibitor olaparib (OLA1) or ± 10 µM olaparib (OLA10) determined by direct counting over time. (**b**) Number of U373 (left; *n* = 12–16) and U373-R (right; *n* = 12–16) cells over time upon treatment ± 1 µM olaparib ± 300 µM TMZ300. (**c**) Clonogenic assays of U373 and U373-R cells that were pretreated for 72 h with increasing concentrations of TMZ ± OLA1 or OLA10, or TMZ ± 1 µM talazoparib (TAL1) of 10 µM talazoparib (TAL10). Displayed are the plating efficiency of TMZ-untreated cells ± olaparib (left; *n* = 6); the plating efficiency of TMZ-untreated cells ± talazoparib (middle; *n* = 6); and the surviving fractions (right; *n* = 5–6), where the data are normalized to the number of corresponding TMZ-untreated cells. All data are shown as means ± SEM. * *p* < 0.05, *** *p* < 0.005 versus olaparib-untreated corresponding cells; ^##^ *p* < 0.01, ^###^ *p* < 0.005, ns: *p* > 0.05 for whole curve comparison; by two-way ANOVA with Sidak’s post hoc test (**a**–**c** right, **d** right) or one-way ANOVA with Dunnett’s post hoc test (**c** left).

Upon DNA damage by chemotherapeutic agents comprising TMZ, poly (ADP ribose) polymerase (PARP) is recruited to the site of DNA damage where it recruits base excision repair (BER) components for DNA repair [29]. PARP inhibitors (PARPi) have therefore been developed and tested to treat cancer. With respect to GBM, although they can increase the antitumor efficacy of TMZ on sensitive cell lines, PARPi generally have little or no effects on TMZ-resistant lines and tumors [30], even if they have a high brain bioavailability, which is typically the case of veliparib [31,32]. Among PARPi, olaparib singles out by its ability to re-sensitize TMZ-resistant GBM cells to TMZ [33], and a Phase I clinical trial showed good brain biodistribution in GBM patients [34]. Based on the hypothesis that olaparib could act at least in part independently of PARP inhibition, we characterized the metabolic effects of this drug on our U373/U373-R model. Olaparib dose-dependently decreased the numbers of U373 and U373-R cells, with no significant effects up to 1 µM, but pronounced effects at 10 µM (Figure 5a). Although it was not cytotoxic alone, 1 µM olaparib strongly increased the sensitivity of U373 and U373-R cells to 300 µM TMZ, shown by direct cell counting (Figure 5b); PE and clonogenic survival (Figure 5c). TMZ resistance was fully reverted in U373-R cells. A higher dose of 10 µM olaparib was as efficient as 300 µM TMZ to reduce cell numbers, with no additive effects (Figure S3a). Similar effects were observed when using talazoparib, another PARPi (Figure 5c).

**Figure 6:**
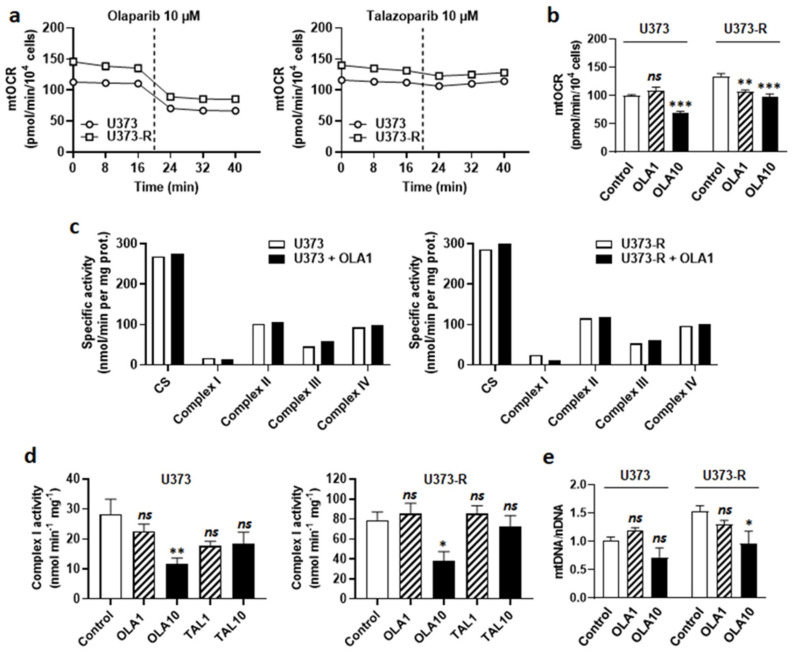
PARP inhibitor olaparib, but not talazoparib, is an ETC Complex I inhibitor. (**a**) U373 and U373-R glioblastoma cells that were treated with 10 µM olaparib or 10 µM talazoparib delivered as a bolus (dotted line) during basal mitochondrial oxygen consumption rate (mtOCR) measurements. Typical Seahorse graphs are shown (*n* = 8). (**b**) Basal mtOCR of cells 5 min after treatment ± olaparib 1 µM (OLA1) or 10 µM (OLA10) (*n* = 5–16). (**c**) U373 (left) and U373-R (right) cells that were treated for 5 min with OLA10. The activities of citrate synthase (CS) and Complexes I-IV of the electron transport chain were measured using enzymatic assays on cell lysates (*n* = 1). (**d**) ETC Complex I activity in U373 (left) and U373-R (right) cell lysates that were treated for 5 min with a single dose of olaparib of talazoparib as indicated (*n* = 4 all). (**e**) The cells were treated for 72 h ± OLA1 or ± OLA10, after which mtDNA/nDNA ratios were determined using RT-qPCR (*n* = 5–8). All data are shown as means ± SEM. * *p* < 0.05, ** *p* < 0.01, *** *p* < 0.005, ns: *p* > 0.05 versus control; by one way ANOVA with Dunnett’s post hoc test (**b**,**d**,**e**).

At the metabolic level, olaparib decreased mtOCR in both U373 and U373-R cell lines (Figure 6a). The response was immediate (Figure 6a) and dose-dependent (Figure 6b), indicating that olaparib interfered with GBM cell respiration independently of PARP inhibition. The fact that PARP inhibition played no role in OXPHOS repression was supported by the observation that talazoparib, which is structurally different from olaparib, did not inhibit GBM cell respiration (Figure 6a). Enzymatic assays on U373 and U373-R cell lysates further revealed that olaparib is a selective ETC Complex I inhibitor (Figure 6c,d). It did not modulate the activities of Complexes II to IV.

Since measuring Complex I activity requires NADH as a substrate, we envisioned the possibility of a direct reaction of olaparib with NADH. Olaparib indeed possesses a protonable nitrogen atom in its imine group and three barely protonable nitrogen atoms in amide functions, allowing, in theory, the production of an iminium intermediate that is capable of reacting with NADH, yielding reduced olaparib (Figure S3b). Comparatively, the imine group of talazoparib is protected by protonable nitrogen atoms in the triazole group. However, nuclear magnetic resonance (NMR) did not detect reduced olaparib when the reaction was attempted in PBS at either pH 7.4 or pH 6.8 (Figure S3c). While this reaction could be more favorable in the catalytic pocket of NADH:ubiquinone oxidoreductase in Complex I, based on its mode of action on PARP [35], olaparib is rather most likely a competitor for NADH at this site. Olaparib at 10 µM, but not 1 µM, further reduced the mtDNA/nDNA ratio (Figure 6e), confirming mitochondrial poisoning at high dose.

### 2.6. Targeting Either Autophagy or Oxidative Metabolism Sensitizes Intrinsically Resistant Human T98G Glioblastoma Cells to TMZ

We finally validated key data using T98G that we considered to be intrinsically resistant to TMZ, as also previously proposed by others [36]. Despite increased MGMT protein expression following selection (Figure S4a), T98G-R and T98G cells were equally responsive to TMZ in clonogenic assays (Figure 1e).

Compared to TMZ-sensitive U373, T98G had a better maximal mtOCR (Figure S4b) and a higher mtDNA/nDNA ratio, which decreased upon treatment with 300 µM TMZ (Figure S4c). These features recapitulated those of the U373-R cells (Figure 4a–c). Furthermore, CQ (10 µM) very efficiently enhanced the response of T98G cells to TMZ, as observed by direct cell counting (Figure S4d), PE, and clonogenic survival assays (Figure S4e). Furthermore, 10 µM bafilomycin A1 combined to 150 µM TMZ fully inhibited T98G cell replication (Figure S4f). In clonogenic assays, ETC Complex III inhibition with 10 µM antimycin A also sensitized T98G cells to TMZ (Figure S4g). Finally, 10 µM olaparib, but not 10 µM talazoparib, decreased T98G mtOCR (Figure S4h) by selectively inhibiting ETC Complex I (Figure S4i,j). It decreased the mtDNA/nDNA ratio (Figure S4k). Although it had no effect on the cell numbers in combination with 300 µM TMZ (Figure S4l), 1 µM olaparib strongly decreased T98G clonogenic survival (Figure S4m).

## 3. Discussion

This study was based on the hypothesis that metabolic alterations could participate in the resistance of human GBM cells to TMZ. To initially minimize background variability, we generated isogenic TMZ-sensitive and TMZ-resistant U373 and U373-R cells that served as main models. To then take into account genetic variability, all of the major findings were tested and confirmed in TMZ-resistant T98G human GBM cells. Compared to sensitive cells, resistant cells had fitter mitochondria with higher OXPHOS activities. Among the tested interventions, we report that (i) the MGMT inhibitor O6BG and PARPi olaparib and talazoparib inhibited the clonogenic survival of human GBM cells independently of their sensitivity to TMZ, (ii) the autophagy/mitophagy inhibitor CQ selectively re-sensitized U373-R cells to TMZ, and (iii) the ETC Complex III inhibitor antimycin A had unpredictable effects (Table S2).

Generating therapy-resistant human cancer cells faces a plethora of commercial cell lines originating from advanced cancers in patients having received one or several lines of therapy. This most likely explains the intrinsic resistance to TMZ of T98G among many other human cell lines [36], leaving us with the U373/U373-R model for initial material. For cells in suspension, the IC_50_ of TMZ has been estimated to be around 100–150 µM for U373 and between ~500 and ~1600 µM for T98G cells [36]. However, in clonogenic survival assays we report IC_50_′s of ~270 and ~240 µM, respectively. After selection, the values were increased in U373-R but not in T98G-R cells, allowing us to consider T98G cells as intrinsically resistant. Therefore, we used the U373/U373-R model for data discovery and T98G cells for validation. Of note, the U373 MG cell line that we used was the authentic one from the original lab in Uppsala, not the discontinued U-251-like line from ATCC [37].

Paired analyses of U373/U373-R cells and T98G/T98G-R cells revealed that a chronic treatment with TMZ increased MGMT protein expression but without increasing resistance to TMZ in the T98G/T98G-R model. Two possible interpretations are that either (i) MGMT does not participate in TMZ resistance in our models, or (ii) MGMT expression can exceed cell needs for DNA repair. Our data support the second proposal, as in clonogenic assays MGMT inhibitor O6BG alone killed more efficiently GBM cells with low MGMT expression, and O6BG had more than additive effects with TMZ (Figure 2 and Table S2). However, the relationship may not be direct. Indeed, while in clinical settings it is well known that MGMT promoter methylation positively correlates with a longer overall survival of patients that are treated with TMZ [12], there is no strict correlation between the level of MGMT protein expression and patient outcome [38]. On the one hand, this suggests that the level of MGMT promoter methylation could reflect epigenetic changes that are more extended than those controlling MGMT expression. On the other hand, it also suggests that O6BG could act beyond MGMT enzymatic repression, and/or that MGMT could control cancer cell metabolism. Accordingly, our observation that human GBM cells that were treated with O6BG switch from an oxidative to a more glycolytic metabolism revealed that there is a metabolic component to the response to MGMT inhibitors. Whether this glycolytic switch represents an attempt of the cells to resist to TMZ by impairing prodrug activation deserves in vivo evaluation, for example by testing the combined effect of MGMT inhibition and proton transporter inhibitors on the response of orthotopic glioblastomas to TMZ. The BBB is expected to constitute an obstacle.

We further report that TMZ-resistant cells are more oxidative and equipped with fitter mitochondria than TMZ-sensitive cells. This would provide a protective advantage against TMZ-induced cell death in a similar way as it accounts for an acquired resistance to other alkylating agents [25,27,39,40]. Indeed, similar to other alkylating agents, TMZ is a mitochondrial poison that decreased mtOCR and the mtDNA/nDNA ratio in GBM cells. TMZ may damage mitochondria in at least two ways: direct mtDNA methylation [41] and the induction of mtROS production at the ETC [42]. Conversely, protection may be conferred by increasing mtDNA copy number, mitochondria quality, their renewal rate, and/or their antioxidant defenses. Accordingly, we observed a higher mtDNA/nDNA ratio (increased DNA copy number) and higher basal and/or maximal mtOCRs (increased mitochondrial quality) in resistant compared to sensitive GMB cells. Mechanistically, Oliva et al. [41] reported an ETC reorganization that was compatible with increased ETC coupling in TMZ-resistant GBM cells, which comprised decreased Complex I and V and increased Complex II, III, and IV activities, supporting decreased mitochondrial proton leak, decreased mtROS generation, and a significantly increased mtOCR reserve capacity.

The higher mtDNA/nDNA ratio that we observed may be linked to increased mitochondrial turnover, as suggested by the observations that OXPHOS was better preserved in resistant cells and that the autophagy/mitophagy inhibitor CQ preferentially killed resistant cells in clonogenic assays. Inhibiting autophagy in general has been widely studied to treat chemoresistant GBM [43]. Buccarelli et al. [44] linked it to mitochondrial quality control by showing that inhibition of autophagy with quinacrine, a compound that is able to cross the BBB, increased the susceptibility of GBM stem cells to TMZ by triggering ferroptosis. Recently, He et al. [45] further reported that TMZ sequentially triggers mtROS production, forkhead box transcription factor 3a (FOXO3a), the expression of Bcl-2/adenovirus E1B 19-kDa-interacting protein 3 (BNIP3), BNIP3 translocation to damaged mitochondria, and their recycling though mitophagy. Thus, mitophagy in particular could be an interesting target to eradicate TMZ-resistant GBM cells, but, to our knowledge, no specific mitophagy inhibitor exists today permeating the BBB.

Directly targeting OXPHOS may be an option to circumvent TMZ resistance. In our hands, Complex III inhibition had variable effects depending on the cell line (Table S2), which might be linked to the fact that, depending on the degree of the inhibition, electron transfer through the ETC can be partially or completely blocked. Comparatively, inhibiting Complex I, for example using metformin, has been reported to more consistently sensitize GBM cells to TMZ [46,47]. Inhibiting Complex I would block ETC electron load by NADH, but not by FADH2, thus sparing part of cell respiration. In other words, Complex III inhibition would mimic the Warburg Effect and protect GBM cells against apoptosis, whereas Complex I inhibition would sensitize them to apoptosis, as previously reported [46,47].

A main finding of our study is that PARPi olaparib, but not talazoparib, is an ETC Complex I inhibitor. Both olaparib and talazoparib were directly cytotoxic, independently of TMZ resistance (Table S2). PARPi have been extensively studied for GBM treatment [35]. Among them, olaparib and veliparib single out as they cross the BBB in patients (talazoparib does not cross the BBB) [31,32,34,48], but only olaparib is known for its capacity to re-sensitize resistant GBM cells to TMZ [33]. By analogy to metformin [46], we propose that the ability of olaparib to inhibit Complex I explains this difference. Our data clearly show that olaparib, but not talazoparib, inhibits Complex I in human GBM cells, ruling out a PARPi class effect. ETC inhibition by olaparib was selective for Complex I and immediate. Since the core component of Complex I is NADH:ubiquinone oxidoreductase and because olaparib is a NAD analogue, we propose that olaparib can occupy and block the catalytic site of the enzyme. There, it would likely act as a competitor for NADH. Re-sensitization of resistant GBM cells to TMZ by olaparib would thus combine OXPHOS inhibition and inhibition of DNA repair.

## 4. Materials and Methods

### 4.1. Cells and Cell Culture

U373 MG (Uppsala) human GBM astrocytoma cells (Sigma-Aldrich, St. Louis, MO, USA, Overijse, Belgium; catalogue #08061901) and T98G human GBM cells (ATCC, Manassas, VA, USA; catalogue #CRL-1690) were routinely cultured in RPMI1640 containing 2 mM GlutaMAX and 11 mM glucose (Life Technologies, Merelbeke, Belgium; catalogue #61870–036), supplemented with 10% fetal bovine serum (FBS) in a humidified incubator at 37 °C, 5% CO_2_. U373-R and T98G-R cells were obtained by treating parental U373 and T98G wild-type cells with increasing concentrations of TMZ for 72 h, starting from 0.1 µM up to 150 µM, with increments of 1.5×, allowing time to recover proliferation between the doses. After selection, the paired cell lines were profiled at Eurofins Genomics (Auderghem, Belgium) with a STR test. To maintain the resistant phenotype, selected cells were weekly exposed to 150 µM TMZ for 72 h.

To modulate pHe, we used a reconstituted medium (Invitrogen, catalogue #51800-019) without sodium bicarbonate, with 2 mM glutamine, 10 mM zwitterionic pH buffer 3-(N-morpholino)-propanesulfonic acid (MOPS, Sigma-Aldrich) ± HCl or NaOH 10% to adjust extracellular pH at 6.8, 7.4 or 8.0, and 10% FBS. The cells were cultured in a humidified incubator at 37 °C receiving filtrated room air.

### 4.2. Drugs and Treatments

Where indicated, the cells were treated with the indicated doses of TMZ (Sigma-Aldrich, catalogue #T2577), MGMT inhibitor O6-benzylguanine (O6BG; Sigma-Aldrich, catalogue #B2292), autophagy inhibitor chloroquine diphosphate (CQ; Sigma-Aldrich, catalogue #C6628), autophagy inhibitor bafilomycin A1 (Sigma-Aldrich; catalogue #SML1661), electron transport chain (ETC) Complex III inhibitor antimycin A (Sigma-Aldrich; catalogue #A8674), poly (ADP-ribose) polymerase inhibitor (PARPi) olaparib (Selleckchem, Huissen, The Netherlands; catalogue #S1060), PARPi talazoparib (Selleckchem; catalogue #S7048), or a combination thereof. The treatment times are indicated in figures and/or figure legends. Unless indicated otherwise, all of the other drugs were from Sigma-Aldrich. In the control experiments, cells received an equal amount of vehicle (DMSO).

### 4.3. Cell Number

The cells were treated as indicated, after which the cell numbers were determined using a SpectraMax i3 spectrophotometer that was equipped with a MiniMax imaging cytometer (Molecular Devices, Munich, Germany). Automatic counts on transmitted light images were captured at the indicated time points.

### 4.4. Clonogenicity

For clonogenic assays, a range of 100 to 400 cells were seeded in 6-well plates and allowed to settle overnight. For every experiment, a control plate was seeded to obtain the plating efficiency (PE). After 72 h of treatment ± test compound(s), media were replaced by fresh media. After colony formation, cells were fixed and stained with 0.5% crystal violet in a 10% ethanol solution for 30–60 min, washed with water, air dried, and counted. The results are expressed as surviving fraction (SF), where SF = #colonies/PE.

### 4.5. Western Blotting

Western blotting was performed as previously described [49]. The primary antibodies were mouse monoclonals against human MGMT (Santa Cruz Biotechnology, Heidelberg, Germany; catalogue #sc-56157) and against β-actin (Sigma-Aldrich; catalogue #A5441). The secondary antibody was an HRP-conjugated goat anti-mouse (Jackson ImmunoResearch, Huissen, The Netherlands; catalogue #115-035-003). The staining was revealed and images were acquired on an Amersham Imager 600 (GE Healthcare, Diegem, Belgium). The Image J software version 1.8.0 (NIH, Bethesda, MD, USA) was used for quantification.

### 4.6. Seahorse Oximetry and pH-Metry

The basal and maximal oxygen consumption rates (OCRs) and extracellular acidification rates (ECARs) were determined using the XF cell mito stress kit (Agilent Technologies, Diegem, Belgium) on a Seahorse XF96 bioenergetic analyzer (Agilent Technologies), according to manufacturer’s recommendations. Briefly, for experiments without concomitant TMZ treatment, 10,000 cells per well were plated on XF96 culture plates 24 h before experiments in RPMI1640 medium containing 2 mM GlutaMAX and 11 mM glucose (Life Technologies; catalogue #61870-036) that was supplemented with 10% FBS. Sequentially, basal OCR was acquired without treatment; maximal OCR after mitochondrial potential disruption using 1 µM ionophore carbonyl cyanide-4-(trifluoromethoxy) phenylhydrazone (FCCP) and non-mitochondrial OCR after the addition of 0.5 µM Complex I inhibitor rotenone together with 0.5 µM Complex III inhibitor antimycin A. Mitochondrial OCRs (mtOCRs) were calculated by subtracting non-mitochondrial OCRs from the corresponding basal and maximal OCRs.

The fuel dependency and capacity were determined in the same conditions but using the Seahorse XF mito fuel flex test kit (Agilent Technologies). Briefly, the fuel dependency was determined by treating the cells with either 20 µM oxidative glucose metabolism inhibitor UK5099, 20 µM glutaminolysis inhibitor BPTES or 30 µM lipolysis inhibitor etomoxir, followed by the simultaneous injection of the 2 other inhibitors. The fuel dependency corresponds to the difference between the basal mtOCR and basal mtOCR with targeted pathway inhibitor (Δ basal mtOCR). The fuel capacity was obtained by first injecting 2 inhibitors and then the inhibitor of the targeted pathway. It represents the maximal capacity of the cells to use a given oxidative fuel when alternative anaplerotic pathways are blocked.

For experiments with TMZ, the protocol was similar, except that 1500 to 3000 cells per well were plated 72 h before experiments, which allowed us to obtain the same final confluence than for TMZ-untreated cells. On the day of analysis, the culture media were replaced by DMEM containing 10 mM glucose, 2 mM glutamine, and 1.85 g/L NaCl, pH 7.4. The cells were incubated for 1 h in a CO2-free incubator before analysis.

Using the above protocols, olaparib or talazoparib were delivered to the cells as a bolus through an injection port A of the Seahorse device.

All of the data were normalized to cell numbers that were measured right before oximetry and pH-metry on a SpectraMax i3 spectrophotometer equipped with a MiniMax imaging cytometer.

### 4.7. Glycolytic Efficiency

Glucose and lactate concentrations in deproteinized cell supernatants were measured using specific enzymatic assays on an ISCUSflex CMA600 analyzer (Aurora Borealis, Schoonebeek, The Netherlands), as previously described [50]. Glucose consumption and lactate production rates were calculated and normalized by total protein content using the Bio-Rad protein assay (Bio-Rad Laboratories, Inc., Temse, Belgium; catalogue #5000006).

### 4.8. Mitochondrial DNA Content

Mitochondrial DNA (mtDNA) copy number was determined following a previously disclosed procedure [24]. The mtDNA content was normalized to nuclear DNA (nDNA) content [51].

### 4.9. Activities of Electron Transport Chain Complexes

The enzymatic activities of ETC complexes I-IV were determined following the protocol of Spinazzi et al. [52] adapted by Aleardi AM et al. [53]. They were measured on a Spectramax M2e spectrophotometer (Agilent Technologies) in whole cell lysates 5 min after the addition of olaparib or talazoparib as indicated, at 37 °C. For assessing Complex I activity, rotenone was used at a concentration of 50 µM. All of the activities are expressed in nanomoles per minute per milligram of total proteins.

### 4.10. Nuclear Magnetic Resonance

The samples were prepared in PBS (pH 7.4 or 6.8) from a stock solution of olaparib (10 mM in DMSO-*d*_6_) and NADH (10 mM in PBS pH 7.4 or 6.8). The final concentration of DMSO-*d*_6_ was 5%. All spectra were acquired at 310 K on a Bruker (Kontich, Belgium) Ascend Avance III 600 MHz system equipped with a broadband cryoprobe. Water signal suppression was achieved using an excitation-sculpting scheme. A total of 32 scans were collected for each experiment sample.

### 4.11. Statistics

All of the data are expressed as means ± SEM. Note that the error bars are sometimes smaller than the symbols. *n* refers to the total number of replicates per condition. The data were analyzed using GraphPad Prism 9.2.0 (San Diego, CA, USA). Student’s *t*-test, one-way ANOVA with Dunnett’s or Sidak’s multiple comparison post hoc test, and two-way ANOVA were used where appropriate. *p* < 0.05 was considered to be statistically significant.

## 5. Conclusions

We report that TMZ-resistant human GBM cells are equipped with more oxidative mitochondria than their TMZ-sensitive counterparts. These fitter mitochondria directly participate in TMZ resistance as OXPHOS inhibition modulates sensitivity. In vitro, targeting ETC Complex I is an option to eradicate TMZ-resistant cells, with olaparib, similar to metformin, acting as a BBB-permeable ETC Complex I inhibitor. Targeting the mitochondrial turnover/quality control is an alternative option that is achievable with autophagy inhibitors, which could be refined by the development of BBB-permeable inhibitors of mitophagy and/or of mitochondrial biogenesis.

## Data Availability

Data is contained within the article or Supplementary Materials.

## Supplementary Materials

The following are available online at https://www.mdpi.com/article/10.3390/ijms222111938/s1. Reference [54] is cited in the Supplementary Materials.
