# Supplementary material for: Olaparib Is a Mitochondrial Complex I Inhibitor That Kills Temozolomide-Resistant Human Glioblastoma Cells
Source: Int J Mol Sci. 2021 Nov 3;22(21):11938. doi: 10.3390/ijms222111938 (PMC8584761; doi:10.3390/ijms222111938)
Supplement: Supplementary file 1 [file ijms-22-11938-s001.zip › ijms-1410211-supplementary.pdf]

Olaparib is a mitochondrial Complex I inhibitor that kills temozolomide-resistant human glioblastoma cells

Luca X Zampieri, Martina Sboarina, Andrea Cacace, Debora Grasso, Léopold Thabault, Loïc Hamelin, Thibaut Vazeille, Elodie Dumon, Rodrique Rossignol, Raphaël Frédérick, Etienne Sonveaux, Florence Lefranc, Pierre Sonveaux

SUPPLEMENTARY FIGURES

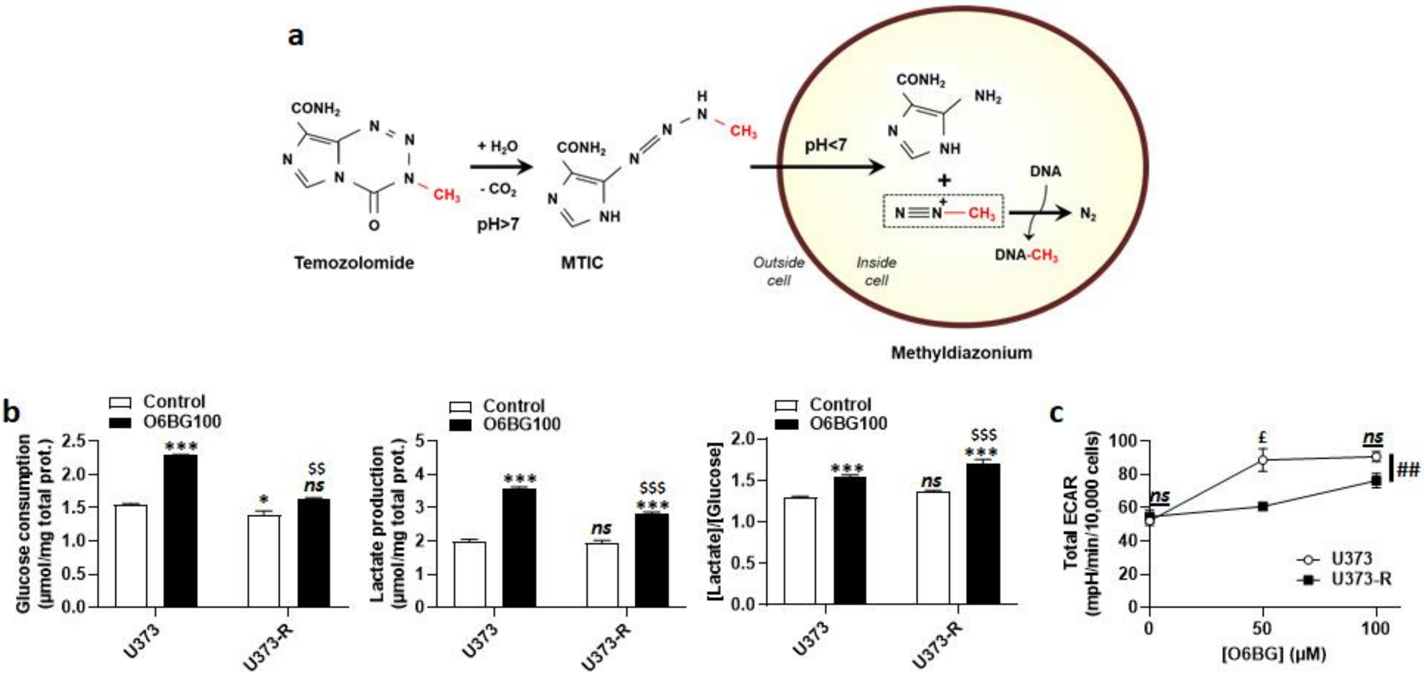

Supplementary Figure S1. MGMT inhibitor O6-benzylguanine promotes glycolysis and extracellular acidification in human glioblastoma cells. a TMZ is a prodrug. At pH > 7, TMZ is extracellularly converted to monomethyl-triazeno-imidazole-carboxamide (MTIC), which crosses cell membranes. At an intracellular pH < 7, MTIC further breaks down to produce methyldiazonium, a highly reactive alkylating agent that methylates DNA. Cartoon adapted from Fukushima *et al.* [54]. **b** Glucose consumption (*left*; $n = 3$) and lactate release (*middle*; $n = 3$) by U373 and U373-R human glioblastoma cells measured after 72 h culture ± 100 µM O6-benzylguanine (O6BG). The right graph represents the glycolytic efficiency ([lactate]/[glucose]; $n = 3$). All data were normalized to total protein content. **c** Cells were pretreated for 72 h with increasing concentrations of O6BG, after which their extracellular acidification rates (ECARs) were measured (n = 8-24). All data are shown as means ± SEM. * $p < 0.05$, *** $p < 0.005$, ns: $p > 0.05$ *versus* vehicle-treated U373 cells; $^{\$\$}$ $p < 0.01$, $^{\$\$\$}$ $p < 0.005$ *versus* vehicle-treated U373-R cells; $^{£}$ $p < 0.05$, <u>ns</u>: $p > 0.05$ *versus* corresponding U373 cells; $^{\#\#}$ $p < 0.01$ for whole curve comparison; by one-way ANOVA with Tukey's post-hoc test (b) or two-way ANOVA with Dunnett's multiple comparison test (c).

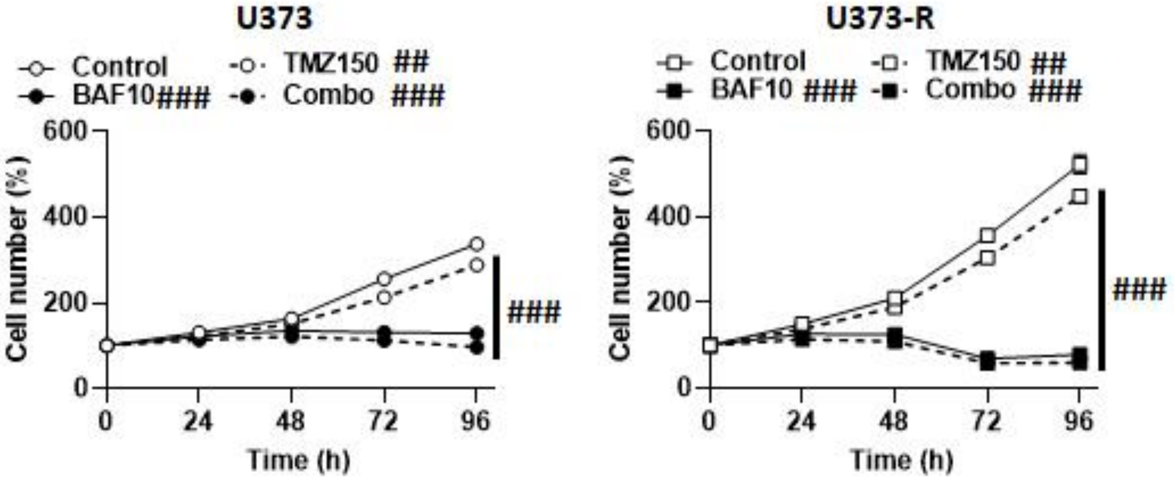

Supplementary Figure S2. ***In vitro*, autophagy inhibitor bafilomycin A1 is a good alternative to temozolomide against U373 human glioblastoma cells.** Number of U373 (*left; n* = 4-8) and U373-R (*right; n* = 4-8) cells over time upon treatment ± 10 nM of bafilomycin A1 (BAF10) ± 150 µM TMZ (TMZ150). All data are shown as means ± SEM. # $p < 0.05$, ## $p < 0.01$, ### $p < 0.005$ for whole curve comparison by two-way ANOVA.

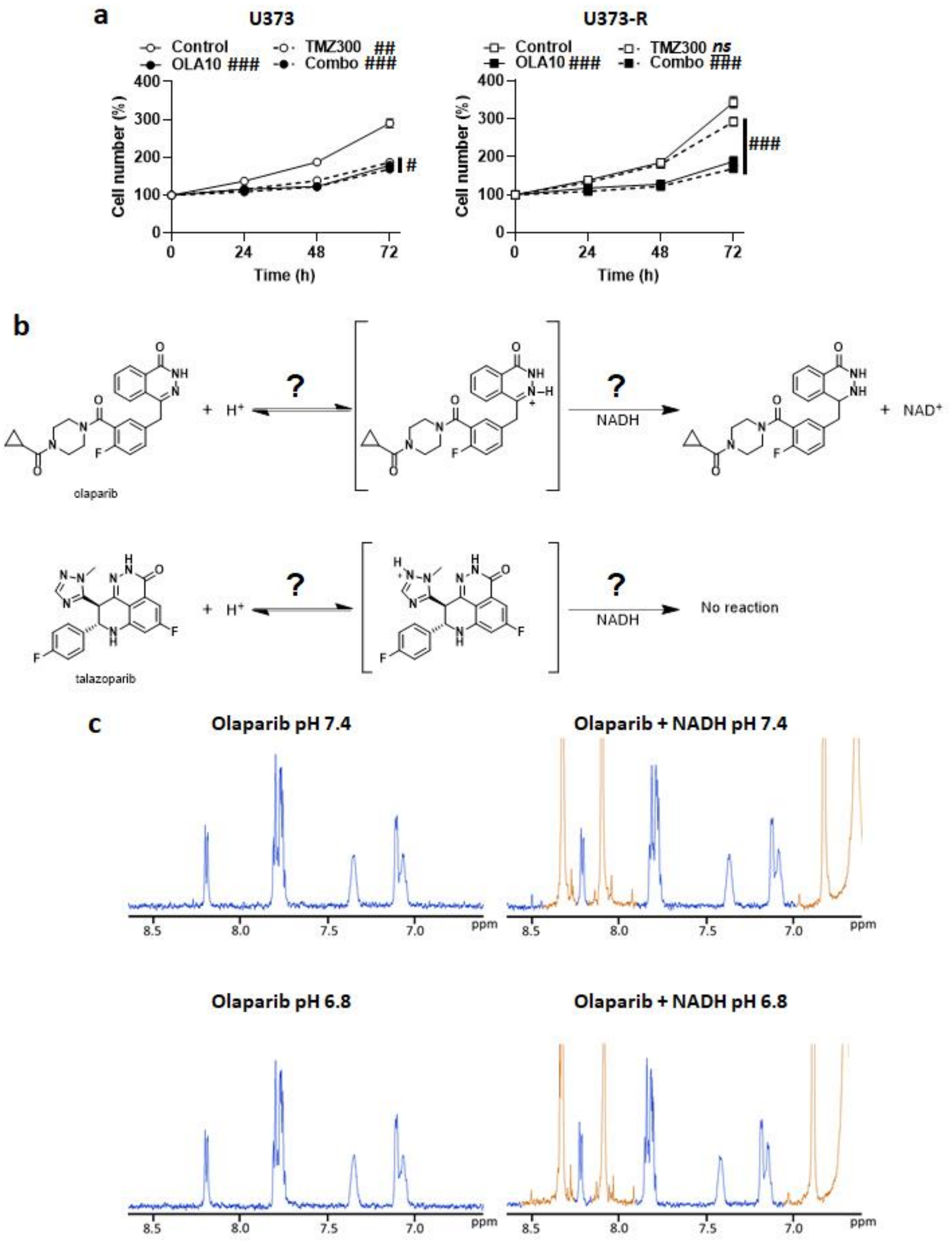

Supplementary Figure S3. PARP inhibitor olaparib represses the expansion of both temozolomide-sensitive and temozolomide-resistant human glioblastoma cells. a Number of U373 (*left; n* = 4-8) and U373-R (*right; n* = 4-7) cells over time upon treatment ± 10 µM olaparib (OLA10) ± 300 µM TMZ (TMZ300). **b** Proposed chemical reactions of olaparib and talazoparib with NADH + H^+. According to the theory, olaparib would be reduced at its imine group, whereas talazoparib reduction would be unfavorable due to protonation of the triazole ring, where the positive charge would be distributed. **c** Representative ^{1}H NMR spectra avquired 45 min after the incubation of 200 µM olaparib (blue) ± 2 mM NADH (orange) in PBS at pH 7.4 or pH 6.8, 37°C. All data are shown as means ± SEM. $^{\#}$ $p < 0.05$, $^{\#\#}$ $p < 0.01$, $^{\#\#\#}$ $p < 0.005$, ns: $p < 0.05$ for whole curve comparison by two-way ANOVA (a).

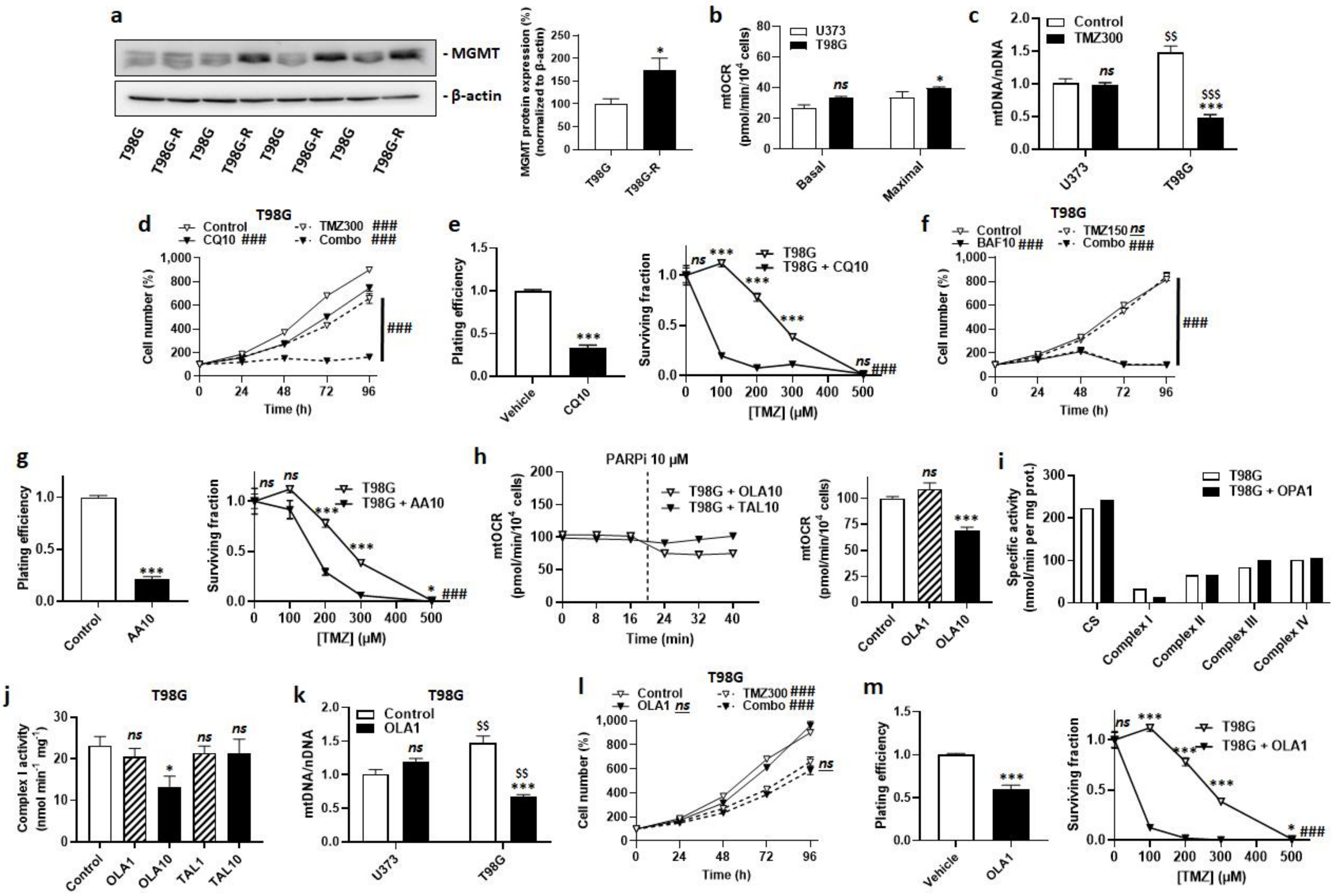

Supplementary Figure S4. Metabolic resentitization of naturally TMZ-resistant T98G human glioblastoma cells. **a** O6-methylguanine DNA methyltransferase (MGMT) and β-actin (loading control) protein expression determined by western blotting in U373 and U373-R glioblastoma cells, with pictures on the left and quantification on the right (*n* = 4). **b** Basal and maximal mitochondrial oxygen consumption rates (mtOCRs) of U373 and T98G glioblastoma cells (*n* = 8-15). **c** Cells were treated for 72 h ± TMZ300, after which mtDNA/nDNA ratios were determined using RT-qPCR (*n* = 4-6). **d** Number of T98G cells over time upon treatment ± CQ10 ± TMZ300 (*n* = 4-8). **e** Clonogenic assays of T98G cells pretreated for 72 h with increasing concentrations of TMZ ± CQ10. Plating efficiency of TMZ-untreated cells (*left; n* = 3-5). Surviving fractions (*right; n* = 5-6), where data are normalized to the number of corresponding TMZ-untreated cells. **f** Number of T98G cells over time upon treatment ± BAF10 ± TMZ150 (*n* = 4-8). **g** As in (e), but using 10 µM of Complex III inhibitor antimycin A (AA10; *n* = 3-6). **h** T98G cells were treated with 10 µM olaparib (OLA10) or 10 µM talazoparib (TAL10) added during (*dotted line*) basal mtOCRs measurements. A typical Seahorse graph is shown on the left. Basal mtOCR of cells 5 min after treatment ± olaparib at 1 µM (OLA1) or 10 µM is shown on the right (*n* = 5-13). **i** T98G cells were treated for 5 min with 10 µM olaparib (OLA10). Activities of citrate synthase (CS) and Complexes I-IV of the electron transport chain were measured using enzymatic assays on cell lysates (*n* = 1). **j** ETC Complex I activity in T98G cell lysates treated for 5 min with a single dose of olaparib of talazoparib as indicated (*n* = 4). **k** U373 and T98G cells were treated for 72 h ± OLA1, after which mtDNA/nDNA ratios were determined using RT-qPCR (*n* = 4-6). **l** As is (d), but using OLA1 (*n* = 4-8). **m** As in (e), but using OLA1 (*n* = 3-6). All data are shown as means ± SEM. * $p < 0.05$, *** $p < 0.005$, *ns*: $P > 0.05$ *versus* corresponding wild-type cells; $^{\#}$ $p < 0.05$, $^{\#\#\#}$ $p < 0.005$, <u>*ns*</u>: $p > 0.05$ for whole curve comparison; $^{\$\$}$ $p < 0.01$, $^{\$\$\$}$ $p < 0.005$ compared to untreated U373 cells; by Student's *t* test (a, b, e *left*, f *left*, k *left*), one-way ANOVA with Tukey's post-hoc test (c, h *right*, j, k) or two-way ANOVA (f) with Sidak's post-hoc test (d, e *right*, g *right*, m *right*).

SUPPLEMENTARY TABLES

Supplementary Table S1. Short tandem repeat (STR) profiles

STRs	U373	U373-R	T98G	T98G-R
**D8S1179**		13.0, 16.0		13.0, 14.0
**D21S11**		28.0, 32.2		28.0, 32.2
**D7S820**	8.2, 12.0	8.0, 12.0	9.0, 10.0	9.0, 10.0
**CSF1PO**	11, 12.0	11.0, 12.0	10.0, 12.0	10.0, 12.0
**D3S1358**		17.0, 17.0		16.0, 16.0
**TH01**	7.0, 9.3	7.0, 9.3	7.0, 9.3	7.0, 9.3
**D13S317**	8.0, 8.0	8.0, 8.0	13.0	13.0, 13.0
**D16S539**	12.0, 13.0	12.0, 13.0	13.0	13.0, 13.0
**D2S1338**		17.0, 25.0		19.0, 24.0
**D19S433**		13.0, 14.0		12.0, 12.0
**vWA**	17.0, 18.0	17.0, 18.0	17.0, 20.0	17.0, 20.0
**TPOX**	8.0, 8.0	8.0, 8.0	8.0	8.0, 8.0
**D18S51**		14.0, 14.0		13.0, 16.0
**AMEL**	X, X	X, X	X, Y	X,Y
**D5S818**	12.0, 12.0	12.0, 12.0	10.0, 12.0	10.0, 12.0
**FGA**		20.0, 20.0		21.0, 22.0

Supplementary Table S2. IC_{50} values of TMZ determined from clonogenic assays.

	**IC_{50} of TMZ** (μM)	**SEM** (μM)	***n***	**IC_{50} of TMZ** (μM)	**SEM** (μM)	***n***	***P***
	**U373**			**U373-R**			
**TMZ alone**	269.75	14.43	6	439.06	15.55	6	***
**TMZ + 50 μM O6BG**	12.90	0.63	6	388.09	20.07	6	***
**TMZ + 100 μM O6BG**	14.76	1.60	6	70.75	2.77	5	***
**TMZ at pH 8.0**	290.20	25.47	6	311.55	52.69	5	*ns*
**TMZ + 10 μM CQ**	282.67	16.12	5	173.26	6.36	5	***
**TMZ + 10 μM antimycin A**	435.54	8.30	5	625.98	23.13	5	***
**TMZ + 1 μM olaparib**	55.99	3.43	5	45.54	9.75	5	*ns*
**TMZ + 1 μM talazoparib**	< 50	ND	6	< 50	ND	6	*ns*
	**T98G**			**T98G-R**			***P***
**TMZ**	238.57	10.20	6	344.43	52.91	6	*ns*
**TMZ + 10 μM CQ**	59.97	5.97	6	ND	ND	ND	ND
**TMZ + 10 μM antimycin A**	169.86	4.78	5	ND	ND	ND	ND
**TMZ + 1 μM olaparib**	48.71	4.36	5	ND	ND	ND	ND

Abbreviations: CQ, chloroquine; ND, not determined; O6BG, O6-benzylguanine; TMZ, temozolomide. All data show means ± SEM. *** $p < 0.005$, *ns*: $p > 0.05$ when comparing wild-type *versus* selected cells.

SUPPLEMENTARY REFERENCE

54. Fukushima, T., Takeshima, H. & Kataoka, H. Anti-glioma therapy with temozolomide and status of the DNA-repair gene MGMT. *Anticancer. Res.* **2009**, *29*, 4845-4854.
